# The role and regulation of blebs in cell migration

**DOI:** 10.1016/j.ceb.2013.05.005

**Published:** 2013-10

**Authors:** Ewa K Paluch, Erez Raz

**Affiliations:** 1 Medical Research Council, Laboratory for Molecular Cell Biology, University College London, Gower Street, WC1E 6BT London, UK; 2 Institute for Cell Biology, Center of Molecular Biology of Inflammation, University of Münster, Von-Esmarch-Strasse 56, Münster, Germany; 3 International Institute of Molecular Cell Biology, Warsaw, Poland

## Abstract

Blebs are cellular protrusions that have been shown to be instrumental for cell migration in development and disease. Bleb expansion is driven by hydrostatic pressure generated in the cytoplasm by the contractile actomyosin cortex. The mechanisms of bleb formation thus fundamentally differ from the actin polymerization-based mechanisms responsible for lamellipodia expansion. In this review, we summarize recent findings relevant for the mechanics of bleb formation and the underlying molecular pathways. We then review the processes involved in determining the type of protrusion formed by migrating cells, in particular *in vivo*, in the context of embryonic development. Finally, we discuss how cells utilize blebs for their forward movement in the presence or absence of strong substrate attachment.

**Current Opinion in Cell Biology** 2013, **25**:582–590

This review comes from a themed issue on **Cell adhesion and migration**

Edited by **Carole A Parent** and **Orion D Weiner**

For a complete overview see the Issue and the Editorial

Available online 17th June 2013

0955-0674/$ – see front matter, © 2013 Elsevier Ltd. All rights reserved.

**http://dx.doi.org/10.1016/j.ceb.2013.05.005**

## Introduction

**Figure 1:**
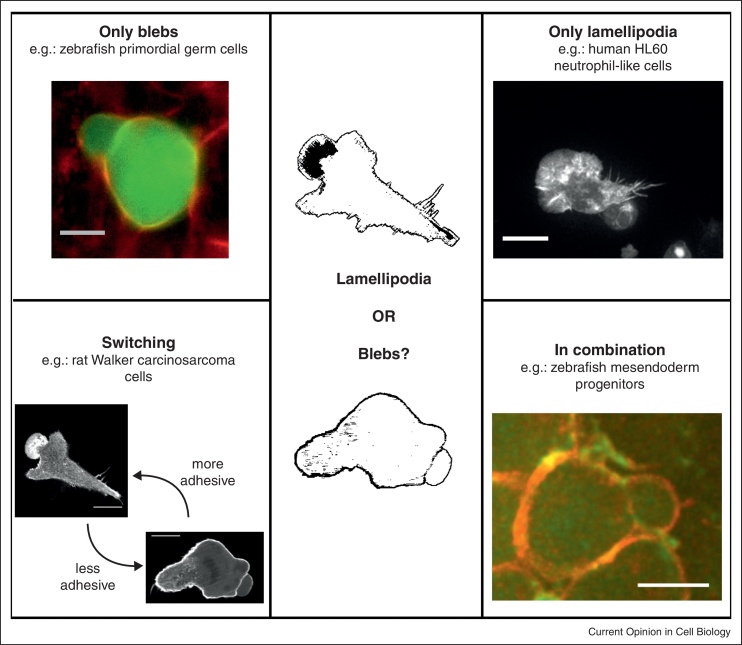
Examples of migrating cells forming exclusively blebs, exclusively lamellipodia, switching between protrusion types or forming both protrusion types in combination. *Top left*: zebrafish PGC expressing a filamentous actin marker (Lifeact-GFP, green) with plasma membranes of all cells labeled in red (mCherry-F globin); image courtesy J. Bandemer. Scale bar: 5 μm. *Top right*: HL60 cell expressing Lifeact-GFP; image courtesy K. Wilson and G. Charras. Scale bar: 10 μm. *Bottom left*: Walker carcinosarcoma cells expressing Lifeact-GFP. Cells are selected for (lamellipodia forming) or against (bleb-forming) adhesion [28^••^]; image courtesy M. Bergert. Scale bars: 10 μm. *Bottom right*: zebrafish mesendoderm progenitor cell expressing a plasma membrane marker (GPI-RFP, red and Lifeact-GFP, green); courtesy A. Diz-Muñoz. Scale bar: 10 μm.

Blebs are hydrostatic pressure and cytoplasmic-flow propelled cellular protrusions that appear as spherical expansions of the membrane, initially devoid of filamentous actin. Long considered a hallmark of apoptosis, blebs have been taking an increasingly central stage in the migration field over the past decade, as it became apparent that they are a widespread leading edge protrusion during cell migration, both in cell culture and *in vivo* (reviewed in [1,2]). Blebbing has been observed in cells moving over two-dimensional (2D) substrates, such as *Dictyostelium discoideum* or *Entamoeba histolytica* [3^•^,4]; however, bleb-based migration is more commonly found in three-dimensional (3D) environments [1]. Whereas certain cell types, such as zebrafish primordial germ cells (PGCs) exclusively utilize blebs during migration [5^••^], other cells, such as fish keratocytes, migrate using actin-driven lamellipodia only [6]. Yet other cell types, such as various metastatic cancer cells, are able to switch between protrusion types, and this plasticity was suggested to help the cells optimize their migration in different environments, for example in the process of tumor dissemination (reviewed in [7,8]). Interestingly, cells migrating *in vivo* during early development often form both lamellipodia and blebs at the same time [9^••^,10^•^]. Blebs can thus form as an alternative to, or in combination with lamellipodia (Figure 1), and are a key protrusion type in 3D migration [11].

In this review we summarize recent findings that provide new insights into the mechanics of bleb formation. We then discuss molecular mechanisms controlling the choice of forming blebs or lamellipodia, particularly in a developmental context. Finally, we discuss potential mechanisms for cell body translocation during bleb-based migration.

## The mechanics of bleb formation

Blebs are powered by intracellular hydrostatic pressure and thus, their formation intimately depends on cellular mechanical parameters. The life cycle of a bleb can be divided into three phases: initiation, growth and retraction (reviewed in [1]). A growing bleb initially appears devoid of filamentous actin [12]. Over time, an actin cortex assembles at the bleb plasma membrane, possibly stalling expansion; the contraction of this newly assembled cortex can then drive bleb retraction [13]. Since in migrating cells, forward movement is often achieved by bleb stabilization that precludes significant retraction, we focus here on the first two phases, initiation and expansion.

### Bleb initiation

**Figure 2:**
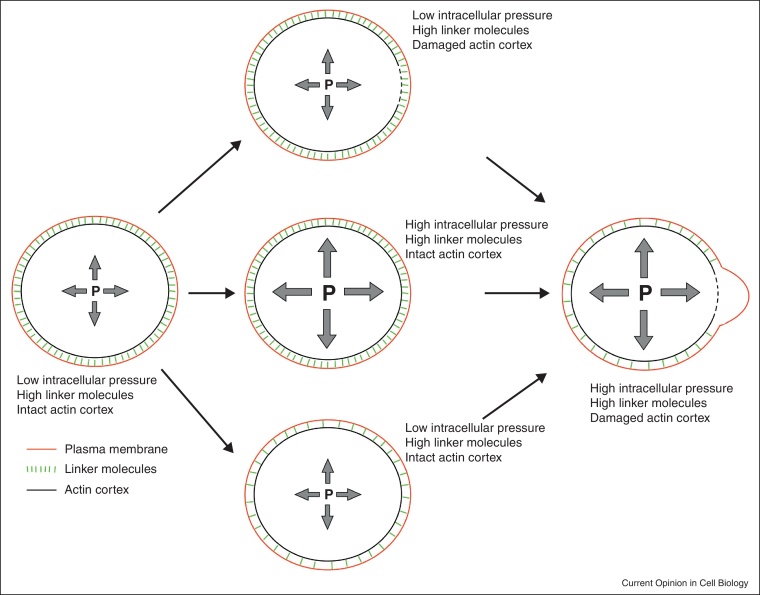
Parameters contributing to bleb formation. Cells whose actin cortex is intact, whose level of cortex–membrane linker proteins is high and which have low intracellular pressure do not form blebs (left cell). Affecting each of the three parameters alone is typically insufficient for the generation of blebs (cells in middle). Blebbing cells are characterized by high myosin-dependent contractility that increases the intracellular pressure, reduced level of linker proteins and/or breaks in the actin cortex at the region where the bleb forms (right cell).

Two mechanisms have been proposed to account for bleb initiation: local decrease in membrane-to-cortex attachment, or local rupture of the cortex itself (Figure 2, reviewed in [1]). Experimentally, it is difficult to distinguish between these two mechanisms, as cortex tears may be small, making them difficult to image [14]. Furthermore, the two mechanisms can act in combination, with a small cortex rupture favoring membrane delamination from the cortex by breaking molecular links connecting the cortex to the membrane, leading to the expansion of a bleb close to the initial tear region [15,16].

Bleb initiation can be induced artificially by affecting each one of the two factors. Local breakage of bonds between the cortex and the membrane could be mechanically triggered by rapid aspiration into a micropipette in rat Walker carcinosarcoma [17], *D. discoideum* [15] and *E. histolytica* cells [4], while local disruption of the actin cortex induced by laser ablation has been shown to result in bleb formation in cultured cells [14], as well as in zebrafish PGCs *in vivo* [18^•^]. Analogously, blebs can be induced by disruption of the actin cortex by local delivery of an actin depolymerizing drug [14,19].

The precise mechanisms responsible for determining the site of bleb initiation during migration are not known. Nevertheless, several observations suggest that asymmetries in the degree of membrane–cortex attachment could play a role in directing blebs to the cell front. For example, in Walker carcinosarcoma cells, the level of the actin–membrane linker ezrin (a member of the ezrin–radixin–moesin (ERM) family) is elevated at the back of the cell, consistent with the idea that in these cells, membrane-to-cortex attachment is reduced at the leading edge, facilitating bleb formation in this part of the cell [20,21^•^,22]. Indeed, an increase in the level or the activity of ERMs is correlated with reduced blebbing in zebrafish germ cells [18^•^], in A375 melanoma cells [23] and in mast cells [24]. In the same direction, as shown in various cell types, weakening the tethering between the membrane and the cell cortex facilitates bleb formation [10^•^,13,18^•^,25]. Together, these observations highlight the reverse correlation between the level of membrane–cortex linker molecules and the potential for bleb formation.

Bleb formation was shown to be critically dependent on the level of myosin contractility [4,5,18^•^,26] and increasing the contractility in cells that normally show rare, or no blebs appears to be sufficient for inducing blebbing [18^•^,27^•^,28^••^]. In the case of zebrafish PGCs, the site of bleb formation has been correlated with local increases of myosin activity, possibly downstream of an increase in intracellular calcium level at the cell front [5^••^]. Local myosin activation could promote cortex tearing, and/or facilitate delamination of the membrane from the cortex [19]. Alternatively, myosin activation could result in a local increase in intracellular pressure that would contribute to the separation of the membrane from the cortex at this location [29^•^]. Such persistent pressure gradients could be maintained by a poroelastic cytoplasm, where intracellular networks and other macromolecular structures interfere with rapid pressure equilibration [30]. Observations in the profusely blebbing melanoma M2 cells, where interfering with bleb formation in one part of the cell does not affect blebbing in other parts of the cell, are consistent with slow pressure equilibration in these cells [29^•^]. In other cells types however, pressure relaxation (by blebs or electroporation) results in a reduction in bleb growth in other locations around the cell perimeter, arguing for fast equilibration of intracellular pressure [4,31^•^]. As the timescale of pressure equilibration strongly depends on the effective cytoplasmic mesh size, the timescale over which pressure gradients equilibrate could strongly vary among different cell types [32].

Finally, localized water uptake, for example, mediated by a polarized distribution of aquaporins could potentially facilitate bleb formation [33]; however, direct experimental evidence supporting such a process in cells employing blebs for their migration is lacking. Electron microscopy images of blebbbing Walker carcinosarcoma cells have revealed potassium-rich pseudo-vacuoles in blebs at the cell's leading edge, suggesting that these structures might facilitate bleb formation by promoting local osmotic swelling [34]. Evaluating this option will require highly accurate volume measurements of live cells, which given the small size of blebs (a few percent of total cell volume), will necessitate the development of novel techniques.

As directionally migrating cells produce blebs oriented toward the migration target, an important question concerns the mechanisms by which bleb formation is biased toward the leading edge of the cell. The correlation between stimulation of cells with chemoattractants and bleb formation (e.g. [35^•^,36]), points at an instructive role of guidance cues in bleb induction. Determining the precise molecular cascade leading from receptor activation to polarization of blebbing activity is an important future research direction.

### Bleb expansion

In the context of cell migration, understanding bleb expansion, the step in which the actual net forward movement of cellular material occurs, is extremely important. Once nucleation has taken place, the expansion of a bleb is thought to be a direct mechanical consequence of intracellular pressure pushing against the plasma membrane. The speed of expansion and the size of the bleb thus depend on cellular physical properties. Two types of physical models have been developed to describe bleb growth: coarse-grained models, which use macroscopic parameters, such as pressure and tension, to account for global cell mechanics without describing the underlying molecular details [16,31^•^,37^•^,38,39]; and molecular models that use computer simulations to derive cell-scale behaviors from microscopic processes [40–42]. While coarse-grained models provide only limited insight into the molecular regulation of cell morphogenesis, microscopic models depend on detailed knowledge of the molecular processes influencing cellular mechanics, processes that are not always experimentally accessible. A combination of both approaches will be required to understand how bleb formation is regulated during cell motility. Nonetheless, the physical models developed over the last few years provide a number of experimentally testable predictions that enhanced our understanding of bleb growth.

A coarse-grained model describing the actin cortex as a contractile actomyosin gel generating hydrostatic pressure in a poroelastic cytoplasm, predicts that as a result of the resistance of the plasma membrane to deformation, a threshold cortical tension for bleb expansion exists [31^•^]. Below this threshold, bleb expansion cannot occur, even in the presence of a rupture in the cortex, or a reduction in membrane-to-cortex attachment. Laser ablation experiments in cells with different cortical tensions support the existence of a threshold tension for bleb expansion [31^•^]. Thus, bleb formation in migrating cells can in principle be controlled at two distinct levels: bleb initiation and bleb expansion (Figure 2). For example, zebrafish PGCs knocked down for the RNA-binding protein Dead end (Dnd) fail to form blebs; laser ablation of the cortex, which bypasses the bleb nucleation step, does not result in bleb formation, indicating that hydrostatic pressure driven bleb expansion is impaired in cells lacking Dnd function [18^•^]. In this case, myosin activation was sufficient to restore blebbing, supporting the notion that myosin contractility and hydrostatic pressure are under the threshold for bleb formation in Dnd-deficient cells.

An additional factor that influences bleb expansion relates to the membrane source for the rapidly inflating bleb. As endosomes are only rarely found in blebs [43] and as the lipid bilayer cannot stretch for more than about 4% without rupturing [44,45], it is assumed that bleb expansion is fuelled by local unfolding of membrane reservoirs at the bleb site and/or larger scale unwrinkling and flow of membrane through the bleb neck. Mechanically, membrane unfolding and flow could resist expansion and effectively slow down bleb growth [37^•^,40,42], potentially constituting a factor in the regulation of bleb growth. Thus far, very little experimental evidence exists concerning the source of membrane and the regulation of membrane supply into the growing bleb. Future investigations of membrane dynamics and tension during bleb growth will be required to address this important issue.

## Control of bleb formation

Whereas some cell types appear to generate exclusively either actin polymerization-based protrusions or blebs driven by actomyosin contractility [5^••^,6], other cells are capable of switching the mode of protrusion formation in response to properties of the environment and intracellular signaling [27^•^,28^••^,46,47]. Three main factors have been proposed to control the type of protrusion formed by a cell: actomyosin contractility, actin polymerization and substrate adhesiveness [48]. Additionally, membrane-to-cortex attachment plays a central role in controlling the ability of cells to form blebs (see above). Together, the combination of enhanced intracellular hydrostatic pressure, reduced level of cortex–membrane protein linkers and breaks in the actin cortex favors local separation of the membrane from the cortex and the nucleation of a bleb (Figure 2). Consistently, increasing actin polymerization favors the generation of actin–driven lamellipodia, while reducing it is correlated with an increase in blebbing [28^••^,49,35^•^].

Modulation of these parameters, individually or in combination, can dictate the specific type of protrusions migrating cells form. For example, in the course of zebrafish gastrulation, noncanonical Wnt signaling controls the balance between amoeboid, bleb forming cell behavior and mesenchymal migration to facilitate effective convergence-extension movements of lateral mesendoderm progenitors [50]. Here, the Wnt pathway elevates contractility by activating Rho that in turn activates the Rock protein, thereby inducing myosin light chain phosphorylation and myosin contractility. In parallel, Rock-mediated phosphorylation of Mypt1 inhibits the function of this Myosin phosphatase, thereby maintaining the level of contractility required for efficient morphogenetic movements [27^•^]. These findings suggest that the proportion of mesenchymal versus bleb-driven motility could be regulated at the level of myosin activity. Another parameter that could affect the efficiency of bleb formation is the strength of membrane–cortex attachment. In zebrafish prechordal plate mesendoderm progenitors, which migrate using a combination of blebs and lamellipodia, reducing membrane–cortex attachment increases bleb formation [10^•^]. In zebrafish PGCs, controlling the level of the membrane–cortex linker protein annexin was shown to be important for the acquisition of bleb-based motility [18^•^].

Investigations in melanoma tumor cells that can interconvert their mode of protrusion formation, allowing them to migrate through different types of environments, have identified the regulation of contractility and actin protrusivity as central for the plasticity in the mode of cell migration [51^••^]. In this case, activation of the Rac protein through the guanine exchange factor (GEF) DOCK3 and the GEF-binding domain containing protein NEDD9 signals through WAVE2 to promote mesenchymal migration. Conversely, activation of ROCK results in elevation of myosin contractility that enhances bleb formation, coupled to inhibition of mesenchymal migration through the activation of the Rac GTPase protein (GAP) ARH-GAP22 thereby inhibiting Rac-promoted actin polymerization and mesenchymal-type protrusion formation.

The ability to switch between protrusion types and motility modes has been proposed to facilitate cancer dissemination; switching could indeed allow cells that migrate through complex and changing environments to select the most efficient migration mode for a given environment (reviewed in [8,46,48]). Importantly, for such switching to enhance migration efficiency, it has to occur rapidly, to adapt to the altered environment on timescales relevant for migration. Indeed, Walker carcinosarcoma cells that can form either blebs or lamellipodia during migration, were shown to instantaneously switch between bleb formation on low adhesion micropatterned substrates and lamellipodia formation on highly adhesive substrates [28^••^]. These findings support the idea that switching between protrusion types can occur ‘on the go’, as the cell faces changes in its environment *in vivo*.

## Mechanisms of bleb-based migration

Whereas blebs are found in migrating cells, blebs are also generated as part of other cellular processes, for example during apoptosis and cytokinesis [14,52]. An important open question is thus whether blebs are indeed essential for the migration of cells that form them, or represent a side effect of enhanced cortical contractility [53^•^]. To answer this question, the actual mechanisms of cell body translocation during bleb-based migration need to be investigated.

**Figure 3:**
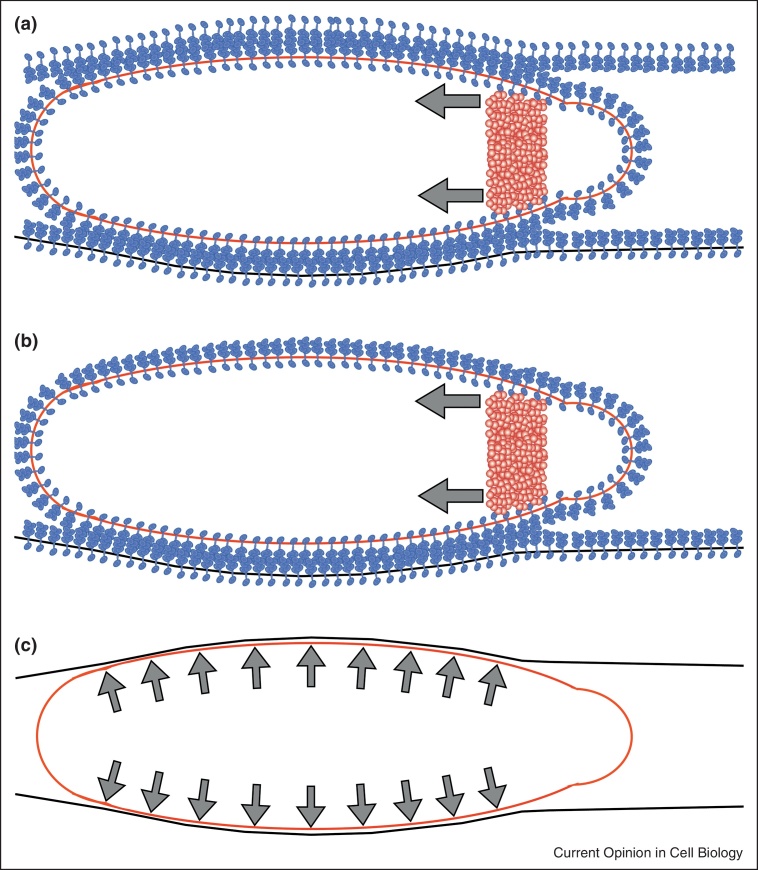
Mechanisms of force transduction in bleb-based migration. Cells can generate traction and move forward by adhering to cells in their environment through cell–cell adhesion or cell–extracellular matrix in three-dimensional or two-dimensional environments **(a and b)** respectively, depicted for cell–cell adhesion, with the arrows indicating actin retrograde flow, or by generating hydrostatic pressure and pressing against cells or structures in their environment (chimneying) **(c)**, arrows indicate pushing forces.

A model proposed by Kardash *et al.* [54] suggests that blebs play a critical role in the forward movement of zebrafish PGCs by way of moving cytoplasm, and thereby the cell's center of mass forward. In this model, actin structures termed ‘actin brushes’ linked to the cell–cell adhesion molecule E-cadherin form at the neck of the bleb and anchor the cells to their environment (Figure 3a). A bleb formed at the cell front can thus force its way forward, while the rest of the cell is fixed, resulting in net transfer of cytoplasm in the direction of migration. A similar mechanism could in principle operate in a 2D environment, if sufficient traction is obtained via adhesion to the substrate, or to neighboring cells (Figure 3b). Migration on 2D substrates has indeed been observed for amoeba using blebs for migration [3^•^,4]. However, other cell types that use blebs in their migration exhibit efficient migration only in 3D environments, suggesting that traction-based translocation may not be sufficient [28^••^].

A traction-independent migration mechanism, termed ‘chimneying’ because of its resemblance of a technique used by alpinists to climb up rock clefts, has been proposed to account for 3D migration of cells in the absence of specific adhesion (Figure 3c). Indeed, leukocytes devoid of receptors that would support adhesion to the extracellular environment were shown to migrate by squeezing and exerting pushing forces perpendicular to the cell boundary [55^••^,56]. A theoretical description of this mechanism suggests that actin polymerizing against the sides of a cell in confinement could produce sufficient pushing forces allowing cell forward movement at velocities higher than the polymerization speed [57]. Alternatively, backwards flows of the actin cortex along the sides of the cell, coupled to friction with the substrate could propel a confined cell forward [58,59]. In this model, friction could result from specific adhesion, or from nonspecific interactions between the cell and the substrate [53^•^,58]. In discontinuous environments, such as in an extracellular matrix network, blebbing could also assist migration by interdigitation of protrusions into gaps within the migration substrate. A theoretical model predicts that such a migration mode would be particularly efficient under very low or no adhesion conditions [60^•^]. Last, a very recent computational model suggests that blebbing cells could move in a swimming-fashion, where net cell body translocation occurs as a result of asymmetric cell shape changes during bleb growth and retraction [61]. Such swimming movements in lamellipodia-forming cells, have been proposed to account for the ability of *D. discoideum* and neutrophils to move up a chemotactic gradient while in suspension [62].

Pushing-based, friction-based, interdigitation-based and swimming-based mechanisms have all been proposed to promote bleb-based motility, but direct experimental evidence for each of these is still scarce. Future studies are required to shed light on the relative contribution of each of these options in promoting cell body translocation of blebbing cells moving in 3D environments. Such investigations will help determining whether 3D confinement supports bleb-based migration by allowing force transduction [58,60^•^] or through its role in constraining cell shape [61].

## Conclusions

Our knowledge of the mechanics and regulation of bleb formation has been considerably broadened in the past five years, and the factors controlling the type of protrusion formed by a migrating cell are now better understood. However, the specific characteristics of bleb-based and lamellipodia-based migration, and the advantages associated with each protrusive type are less clear. Several features have been identified as being potentially specific to bleb-based migration. The relatively fast formation of blebs suggests that this protrusion type provides an efficient mean to explore the extracellular environment. Consistently, enhanced blebbing correlates with decreased directional persistence in migration during zebrafish development [10^•^]. In some cases, blebbing motility appears to require less, or no, specific adhesive interactions with the environment [48]. Such a strategy might have facilitated the migration of primitive cells, which had not yet developed means for supporting motility by specifically adhering to their environment.

Interestingly, blebbing is employed in the migration of certain cell types, is not used by others, and can be utilized in combination or interchangeably with other protrusion modes in early development and cancer. Future studies benefiting from the enhanced understanding of the mechanisms of bleb formation will help understand how modulation of cellular and environmental properties (Figure 2) controls the protrusion type formed during migration in specific contexts. It will be interesting to correlate the individual strategies employed by migrating cells with the challenges they face and their evolutionary and developmental history.

## References and recommended reading

Papers of particular interest, published within the period of review, have been highlighted as:

• of special interest
•• of outstanding interest
